# Correction: Alanazi, F.; Alrwaily, M. Assessing Physical Therapy Knowledge amongst the New Graduates in Saudi Arabia: Competency Examination across the Nation. *Healthcare* 2022, *10*, 579

**DOI:** 10.3390/healthcare10071286

**Published:** 2022-07-12

**Authors:** Fahad Alanazi, Muhammad Alrwaily

**Affiliations:** 1 Department of Physical Therapy, College of Applied Medical Sciences, Jouf University, Sakaka 72388, Saudi Arabia; 2 Division of Physical Therapy, School of Medicine, West Virginia University, Morgantown, WV 26506, USA; muhammad.alrwaily@hsc.wvu.edu

## Supplementary Materials Correction

In the original publication [1], there was a mistake in the Supplementary Materials as published; we would like to remove this part.

The authors apologize for any inconvenience caused and state that the scientific conclusions are unaffected. This correction was approved by the Academic Editor. The original publication has also been updated.
